# Delayed-enhancement cardiac magnetic resonance imaging detects disease progression in patients with mitral valve disease and atrial fibrillation

**DOI:** 10.1016/j.xjon.2023.07.024

**Published:** 2023-08-17

**Authors:** Tari-Ann Yates, Ramya Vijayakumar, Martha McGilvray, Ali J. Khiabani, Nicholas Razo, Laurie Sinn, Matthew R. Schill, Nassir Marrouche, Christian Zemlin, Ralph J. Damiano

**Affiliations:** a Division of Cardiothoracic Surgery, Washington University School of Medicine, Barnes-Jewish Hospital, Saint Louis, Mo; b Department of Cardiology, Tulane University School of Medicine, New Orleans, La

**Keywords:** atrial fibrillation, mitral valve disease, delayed-enhancement magnetic resonance imaging, atrial fibrosis, mitral regurgitation

## Abstract

**Objectives:**

The mechanism by which mitral valve (MV) disease leads to atrial fibrillation (AF) remains poorly understood. Delayed-enhancement cardiac magnetic resonance imaging (DE-MRI) has been used to assess left atrial (LA) fibrosis in patients with lone AF before catheter ablation; however, few studies have used DE-MRI to assess MV-induced LA fibrosis in patients with or without AF undergoing MV surgery.

**Methods:**

Between March 2018 and September 2022, 38 subjects were enrolled; 15 age-matched controls, 14 patients with lone mitral regurgitation (MR), and 9 patients with MR and AF (MR + AF). Indexed LA volume, total LA wall, and regional LA posterior wall (LAPW) enhancement were defined by the DE-MRI. One-way analysis of variance was performed.

**Results:**

LA volume and LA enhancement were associated (r = 0.451, *P* = .004). LA volume differed significantly between controls (37.1 ± 10.6 mL) and patients with lone MR (71.0 ± 35.9, *P* = .020 and controls and patients with MR + AF (99.3 ± 47.4, *P* < .001). The difference in LA enhancement was significant between MR + AF (16.7 ± 9.6%) versus controls (8.3 ± 3.9%, *P* = .006) and MR + AF versus lone MR (8.0 ± 4.8%, *P* = .004). Similarly, the was significantly more LAPW enhancement in the MR + AF (17.5 ± 8.7%) versus control (9.2 ± 5.1%, *P* = .011) and MR + AF versus lone MR (9.8 ± 6.0%, *P* = .020)

**Conclusions:**

Patients with MR + AF had significantly more total and LAPW fibrosis compared with both controls and lone MR. Volume and delayed enhancement were associated, but there was no difference between MR and MR + AF.

## Graphical Abstract

**Figure:**
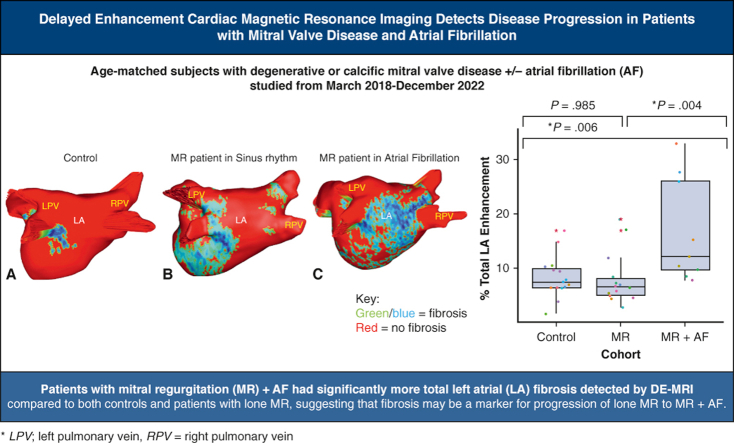

**Figure:**
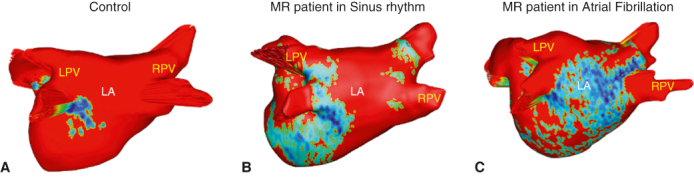
DE-MRI fibrosis maps of (A) control subject (B), lone MR patient (C), MR + AF patient.

**Central Message**

Patients with MR + AF had significantly more LA and regional LAPW fibrosis compared to both controls and lone MR. Volume and wall enhancement were associated with no difference between MR and MR + AF.

**Perspective**

There is a paucity of data using noninvasive delayed-enhancement magnetic resonance imaging to identify and quantify fibrosis as the anatomical substrate for AF in patients with degenerative and calcific MV disease. Patients with MR-induced AF have significantly more LA fibrosis, especially in the LAPW, compared to both controls and lone MR. Fibrosis levels may identify progression of lone MR to MR+AF.

See Discussion on page 303.

Valvular heart disease is the most common etiology of chronic atrial fibrillation (AF) in the world.^1^ Patients referred for the surgical treatment of mitral valve (MV) disease are the most common group to have concomitant AF ablation. Although there are surgical guidelines with class 1 evidence that recommend surgical ablation for symptomatic patients with MV undergoing cardiac surgery,^2^ the mechanisms by which the MV disease leads to AF remain poorly understood.

It has been hypothesized that atrial fibrosis in combination with volume overload is the pathological substrate required to both initiate and sustain AF in patients with MV disease. Several studies have used protein molecular analysis on atrial tissue biopsies to demonstrate that patients with degenerative MV disease and AF had more fibrosis than those with MV disease in normal sinus rhythm (NSR)^3^, ^4^, ^5^, ^6^ and those with lone AF.^7^^,^^8^ Furthermore, one study found more regional atrial fibrosis in the left atrial posterior wall (LAPW) of patients with MV disease and AF than with patients with MV disease in NSR.^5^ This finding is clinically important because the LAPW region has been associated with arrhythmogenesis and incomplete electrical isolation of this area during ablation is a risk factor for atrial tachyarrhythmia recurrence.^9^, ^10^, ^11^

Despite much effort to understand whether AF is the cause or consequence of fibrosis, it is still not well defined how the arrhythmogenic substrate is developed in patients with MV disease, and there are few noninvasive tests that can be used to quantify the potential proarrhythmic substrate. The present study used delayed-enhancement magnetic resonance imaging (DE-MRI), which has been validated by histopathologic assessment, to quantify the degree of left atrial (LA) fibrosis.^12^^,^^13^ DE-MRI involves an intravenous administration of gadolinium contrast that is taken up by the cardiac extracellular matrix and represents the expansion of the extracellular matrix by the formation of fibrosis.^14^^,^^15^ Numerous other studies in the catheter-ablation literature, mostly in patients with lone AF, have shown that DE-MRI enhancement is a marker of fibrosis.^6^, ^7^, ^8^, ^9^, ^10^, ^11^, ^12^, ^13^, ^14^, ^15^, ^16^, ^17^, ^18^

The quantification of the fibrotic LA substrate using DE-MRI has been well studied in the multicenter prospective Association of Atrial Tissue Fibrosis Identified by Delayed Enhancement MRI and Atrial Fibrillation Catheter Ablation (DECAAF I) and Effect of MRI-Guided Fibrosis Ablation Versus Conventional Catheter Ablation on Atrial Arrhythmia Recurrence in Patients With Persistent Atrial Fibrillation (DECAFF II) trials, which found that fibrosis levels before and after catheter ablation in patients with paroxysmal and persistent AF independently predicted postablation AF recurrence and defined an optimal fibrosis targeted approach to catheter ablation. Those patients with a low level of fibrosis preablation had better postablation rhythm outcomes than patients with a greater level of fibrosis.^17^^,^^18^ However, this imaging modality has not been used to identify a similar substrate in patients with MV disease. Therefore, this study used DE-MRI to investigate the effects of degenerative and calcific mitral regurgitation (MR) on LA DE-MRI enhancement (Video 1). It was hypothesized that quantification of LA enhancement would correlate with worsening pathology from control age-matched patients to patients with lone MR, to patients with MR with a history of AF (MR + AF).

## Methods

### Patients

This was a single-center prospective study with enrollment from March 2018 to December 2022. The study was approved by the Washington University School of Medicine in St Louis Institutional Review Board (#201801126, current approval date: October 11, 2022). Informed consent and permission for release of information were obtained from all patients. Control subjects did not undergo surgery, and inclusion criteria for control subjects were the following: age 18 years of age or older, no history of AF or heart disease, and a normal electrocardiogram. Inclusion criteria for patients with MR referred for MV surgery were a diagnosis of degenerative or calcific MV disease with or without AF.

Those who had AF also underwent a concomitant Cox maze IV procedure (CMP-IV) as previously described in the literature.^19^ LA appendage management varied by patient, with closure achieved by amputation with epicardial oversewing, epicardial placement of an AtriClip (AtriCure Inc), or oversewing the appendage from the endocardium.

Exclusion criteria for controls included any history of previous arrhythmia or cardiac surgery, catheter ablation, uncontrolled hypertension, left ventricular ejection fraction <30%, pregnant or breastfeeding, contraindications to MRI, current dialysis or acute kidney injury, or body mass index >40. Exclusion criteria for patients with MR were the same except for prior arrhythmia, history of failed catheter ablation and/or other concomitant valve or cardiac procedures such as coronary artery bypass grafting, aortic or tricuspid valve surgery.

Demographic data from controls were collected using a questionnaire. All clinical characteristics of patients were defined by the adult cardiac Society of Thoracic Surgeons definitions except for LA volume, which was defined by MRI report and duration of MR, which was defined the time difference between the date of surgery and the earliest preoperative transthoracic echocardiogram date, in years, which was obtained from chart review. No subject or patient data were missing. Missing data were ascertained through chart review, contact with patients, and referring physicians. Each patient’s rhythm was captured by electrocardiography before imaging. If applicable, the type of AF was defined by the current 2017 Heart Rhythm Society guidelines.^20^ Longstanding persistent AF was defined as continuous AF >1 year, persistent AF was defined as lasting beyond 7 days, and paroxysmal AF was defined as terminating spontaneously or with intervention within 7 days.^2^

### Cardiac MRI and Atrial Enhancement Quantification

**Figure 1:**
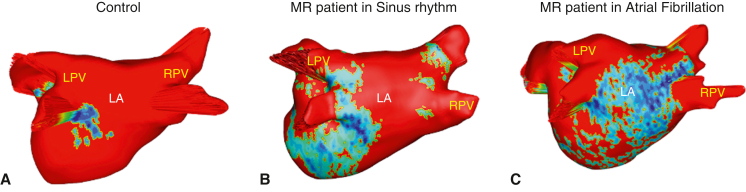
3D LA fibrosis maps of (A) control subjects, (B) patients with lone MR, and (C) patients with MR + AF. Healthy tissue is depicted in *red*, and DE-MRI enhancement is depicted in *blue*, *white*, and *yellow*. *Right* (R) and *left* (L) pulmonary veins (*PVs*) labeled accordingly. *LPV*, Left pulmonary vein; *LA*, left atrial; *RPV*, right pulmonary vein; *3D*, 3-dimensional; *MR*, mitral regurgitation; *AF*, atrial fibrillation.

All subjects underwent a 3-Tesla (T) delayed gadolinium (0.2 mmol/kg) enhancement MRI protocol, with patients undergoing the scan within 30 days of the elective surgery.^17^^,^^18^^,^^21^ DE-MRI defined the anatomy and structure of the atria and pulmonary veins, and quantified the degree of LA fibrosis. Total right atrial fibrosis data are provided, but only the total and regional LA fibrosis analysis has been approved for clinical use. Commercial software (MARREK Inc) approved by the Food and Drug Administration was used for image segmentation and processing to provide 3D LA fibrosis maps and quantification reports, which included LA volume measurements (Figure 1). Gadolinium-enhanced MR angiography was used to delineate the endocardium.

DE-MRI images of the atria were acquired approximately 20 minutes after contrast administration using a 3-dimensional electrocardiographic-gated, respiratory-navigated, and inversion recovery–prepared gradient echo pulse sequence. Inversion preparation was applied to every heartbeat, and fat saturation was performed immediately before data acquisition. Data acquisition was limited to 15% of the averaged cardiac cycle length and was performed during LA diastole. The other scan parameters for DE-MRI of atria at the 3-T scanner were an axial imaging volume with fields of view = 400 × 400 × 150 mm, voxel size = 1.25 × 1.25 × 2.5 mm, repetition time = 3.1 milliseconds, echo time = 1.4 milliseconds, and flip angle of 14°.

**Figure 2:**
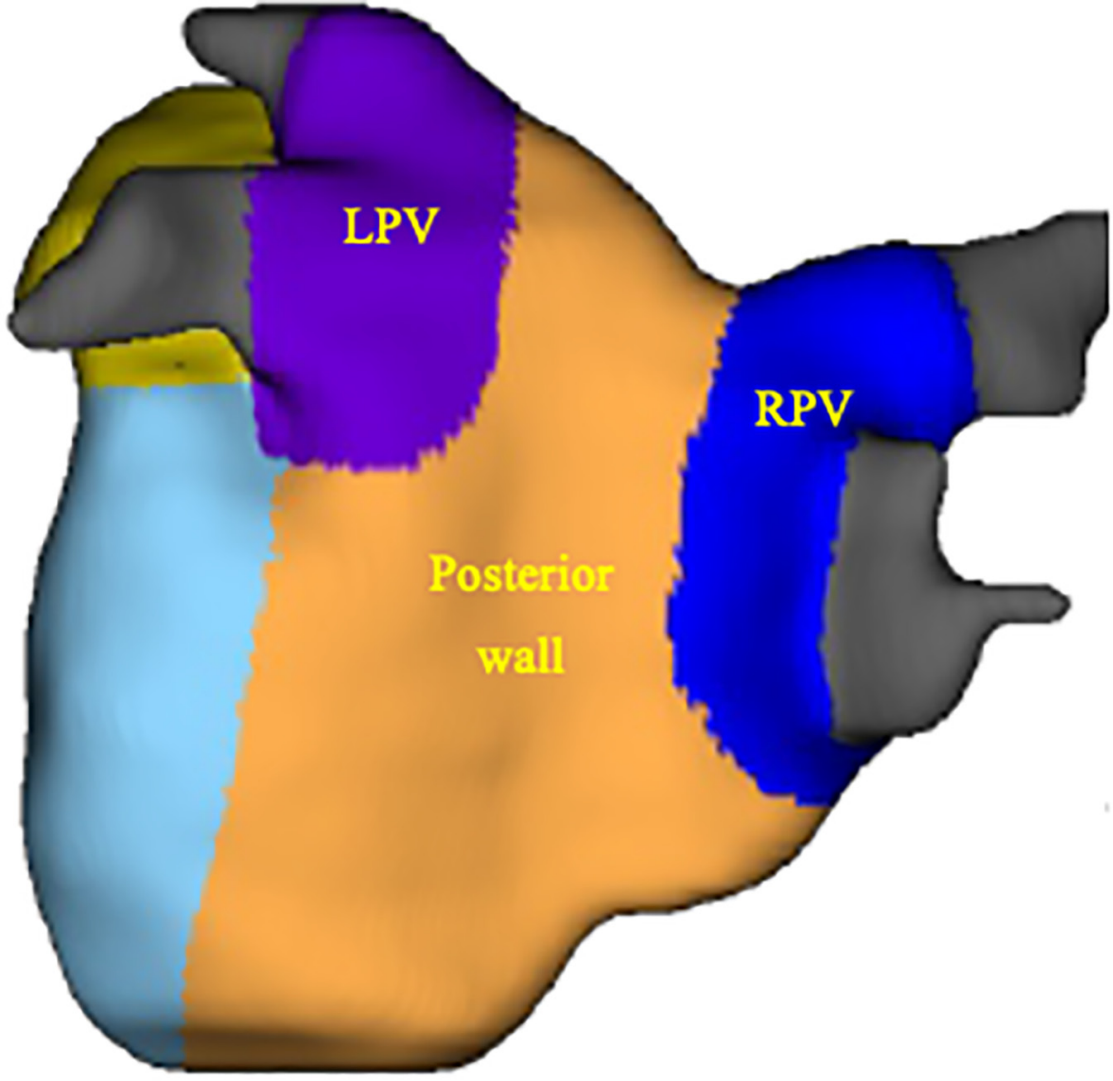
Map of the LA regions. The left atrial posterior wall (*LAPW*) defined summation of the regional enhancement percentage and wall volume of the posterior wall (*orange*), *right* (R, *blue*) and *left* (L, *purple*) pulmonary vein (*PVs*) labeled accordingly. *LA*, Left atrial; *RPV*, right pulmonary vein.

The left atrium 3-dimensional maps of the fibrosis scans derived from the DE-MRI scans that were of good- or fair-quality scans were evaluated and the extent of atrial wall enhancement was categorized using the Utah classification for LA fibrosis staging as follows: Class 1 (<10%), Class 2 (10%-20%), Class 3 (20%-30%), and Class 4 (>30%).^16^, ^17^, ^18^ The LAPW region enhancement was computed, which consisted of the left pulmonary vein, right pulmonary vein, and posterior wall regions from the fibrosis report (Figure 2). It is not possible to anatomically isolate just the PVs from the cardiac MRI analysis.

### Statistical Analysis

Data are reported as mean ± standard deviation unless otherwise stated. For continuous variables comparing 2 groups, a Student *t* test was used to calculate significance. For continuous variables comparing 3 groups, the significance between the observed means was calculated using a one-way analysis of variance test with Tukey post-hoc tests. For categorical variables, a Fisher exact test was used to determine significance unless otherwise stated. The software package used for statistical analysis was IBM SPSS Statistics, Version 28.0.0.0 (IBM Corp).

## Results

### Clinical and Operative Characteristics

**Figure E1:**
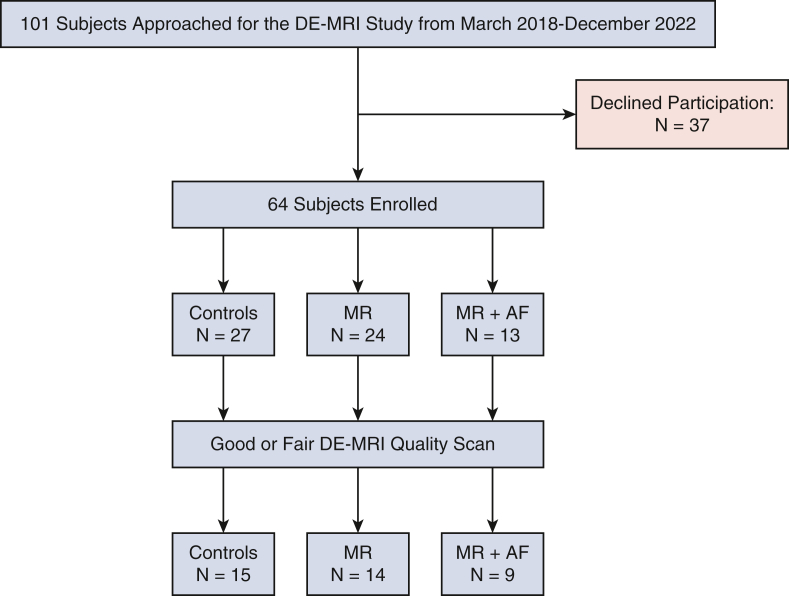
Consolidated Standards of Reporting Trials diagram. Study enrollment. *DE-MRI*, Delayed-enhancement magnetic resonance imaging; *MR*, mitral regurgitation; *AF*, atrial fibrillation.

During the study period, 101 subjects were approached, of whom 63 (63%) were enrolled into the study. Of those 63 subjects, 38 had good- or fair-quality DE-MRI scans that were analyzed, which included 15 age-matched controls, 14 patients with lone MR, and 9 patients with MR + AF (Figure E1). There were no differences in age, sex, race, or baseline comorbidities between the groups (Table 1).

**Table 1:** Clinical characteristics

Characteristics (N = 38)	Controls (N = 15)	MR (N = 14)	MR + AF (N = 9)	*P* value
Age, y	66.1 ± 7.8	60.7 ± 9.8	69.0 ± 7.4	.127
Sex, n (%)				.193
Male	5 (33)	9 (64)	7 (78)	
Female	10 (57)	5 (36)	2 (22)	
Race, n (%)				1.000
White	13 (86)	14 (100)	9 (100)	
Black	1 (7)	0 (0)	0 (0)	
Asian	1 (7)	0 (0)	0 (0)	
BMI, n (%)	28.2 ± 6.4	28.4 ± 4.6	30.7 ± 5.9	.767
HTN, n (%)	5 (33)	5 (36)	7 (78)	.263
DM, n (%)	1 (7)	1 (7)	3 (33)	.277
COPD (moderate/severe lung disease), n (%)	1 (7)	0 (0)	1 (11)	.422
Previous CVA, n (%)	1 (7)	0 (0)	0 (0)	1.000
Previous MI, n (%)	0 (0)	0 (0)	1 (11)	.105
PVD, n (%)	0 (0)	1 (7)	0 (0)	1.000
Smoking history, n (%)	0 (0)	4 (29)	1 (11)	.073

*P* value from one-way analysis of variance for continuous variable and Fisher exact test for categorical variables significant if less than or equal to .05. *MR*, Mitral regurgitation; *AF*, atrial fibrillation; *BMI*, body mass index; *HTN*, hypertension; *DM*; diabetes; *COPD*, chronic obstructive pulmonary disease; *CVA*, cerebrovascular disease; *MI*, myocardial infarction; *PVD*, peripheral vascular disease.

The majority of patients who underwent concomitant MV surgery and CMP-IV had a history of paroxysmal AF (6/9, 67%), and the other 3 patients had a history of nonparoxysmal AF (1 patient had persistent AF and 2 patients had longstanding persistent AF). The mean AF duration of all patients was 2.3 ± 2.5 years.

Two patients had longstanding persistent atrial fibrillation, of whom one had an AF duration of 18 months and Utah class 1 (<10% fibrosis of the total LA wall), and the other had an AF duration of 21 months and Utah class 4 fibrosis (>30% fibrosis of the total LA wall) (Table E1). The only patient with persistent AF had an AF duration of 6 months and Utah class 1 (Table E1). Of the 6 patients with paroxysmal AF, 2 had Utah class 3 (20%-30% fibrosis of the total LA wall) with a duration of 84 months and 60 months. Of the remaining 4 patients with paroxysmal AF, 3 had Utah class 2 (10%-20% fibrosis of the total LA wall), with durations of 1 day, 4 months, and 20 months, and 1 patient had Utah class 1 with a duration of 2 months (Table E1). The 1 patient with an AF duration of 1 day was diagnosed preoperatively the day of surgery and presented in NSR the day of MRI scan.

During the MRI scan, all patients with lone MR were in NSR. Of the patients with a history of AF, 4 were in NSR and 5 were in AF. The majority of the patients who underwent MV surgery had degenerative MV disease and underwent MV repair (Table 2). The severity of MR and preoperative hemodynamic characteristics of patients with lone MR and MR + AF are detailed in Table 2.

**Table 2:** Mitral valve surgery and hemodynamic findings

Variables (N = 23)	MR (N = 14)	MR + AF (N = 9)	*P* value
LA volume, mL/m^2^∗	71.0 ± 35.9	99.3 ± 47.4	.117
LVEF, %†	65.8 ± 7.5	58.9 ± 15.5	.186
MV surgery, n (%)			
Repair	13 (93)	8 (89)	
Replacement	1 (7)	1 (11)	1.000
MR severity, n (%)			
Grade 3	1 (7)	2 (22)	
Grade 4	13 (93)	7 (78)	.270
MR duration, y‡	0.9 ± 1.7	0.3 ± 0.5	.352
TR severity, n (%)			.142
Grade 3	14 (100)	7 (78)	
Grade 4	0 (0)	2 (22)	

*MR*, Mitral regurgitation; *AF*, atrial fibrillation; *LA*, left atrium; *LVEF*, left ventricular ejection fraction; *MV*, mitral valve; *TR*, tricuspid regurgitation; *MRI*, magnetic resonance imaging.

∗ LA volume from MRI patient report.

† LVEF is a Society of Thoracic Surgeons–defined variable. *P* values for Student *t* test for continuous variables or Fisher exact test for categorical variables are significant if less than or equal to .05.

‡ MR duration defined by the time difference between the date of surgery and the earliest preoperative transthoracic echocardiogram date, in years, which was obtained from chart review.

### DE-MRI Characteristics

There were significant differences in the LA volume, total LA wall enhancement, and regional LAPW enhancement between the 3 groups (Table 3). Furthermore, the only patients with Utah class 3 or 4 enhancement were seen in the MR + AF group (Table 3).

**Table 3:** DE-MRI characteristics

MRI characteristics	Controls (N = 15)	MR (N = 14)	MR + AF (N = 9)	*P* value
LA volume, indexed, mL/m^2^	37.1 ± 10.6	71.0 ± 35.9	99.3 ± 47.4	**<.001**
LA wall enhancement, %	8.3 ± 3.9	8.0 ± 4.8	16.7 ± 9.6	**.003**
LAPW enhancement (5%)	9.2 ± 5.1	9.8 ± 6.0	17.5 ± 8.7	**.009**
Utah class, n (%)				.061
1	11 (73)	11 (79)	3 (33)	
2	4 (27)	3 (21)	3 (33)	
3	0 (0)	0 (0)	2 (23)	
4	0 (0)	0 (0)	1 (11)	

*P* values from one-way analysis of variances for continuous variables and Fisher exact test for categorical variables significant if less than or equal to .05. *P* values in bold are statistically significant. *MRI*, Magnetic resonance imaging; *MR*, mitral regurgitation; *AF*, atrial fibrillation; *LA*, left atrium; *LAPW*, left atrial posterior wall; *DE*, delayed enhancement.

### Indexed LA Volume

**Figure 3:**
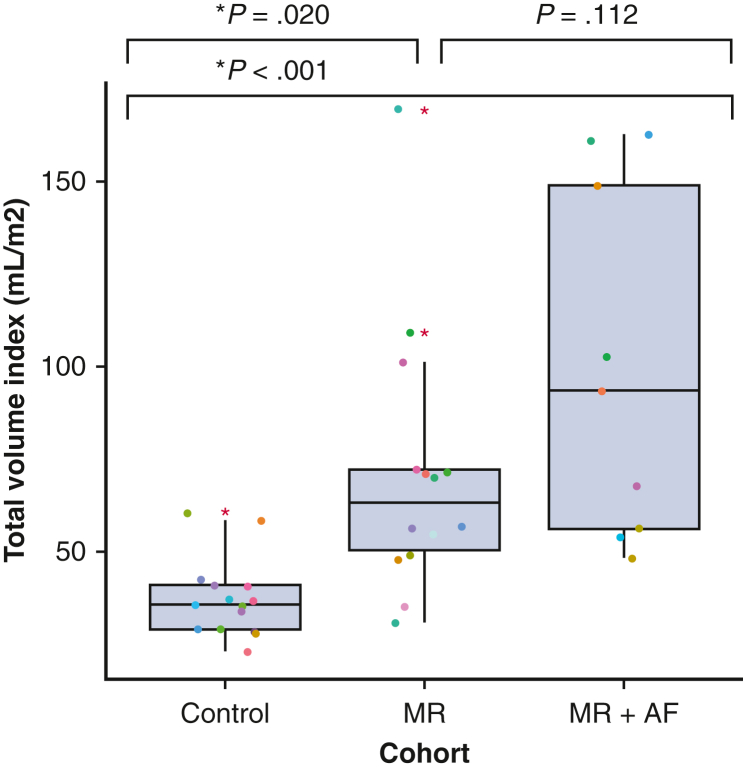
Total LA volume indexed between control patients, patients with lone MR, and patients with MR + AF. In the *box* and *whiskers* plot, the *lower* and *upper borders* of the *box* represent the *lower* and *upper* quartiles (25th percentile and 75th percentile). The *middle horizontal line* represents the median and the *lower* and *upper* whiskers represent the minimum and maximum values of nonoutliers. *Extra dots* represent outliers. *MR*, Mitral regurgitation; *AF*, atrial fibrillation; *LA*, left atrial.

There was a significant difference in the indexed LA volume between control patients versus patients with MR (37.1 ± 10.6 mL vs 71.0 ± 35.9 mL, *P* = .020) and control patients versus patients with MR + AF (37.1 ± 10.6 mL vs 99.3 ± 47.4 mL, *P* < .001). However, there was no significant difference between patients with MR and patients with MR + AF (71.0 ± 35.9 mL vs 99.3 ± 47.4 mL, *P* = .112, Figure 3).

### Total LA Wall Enhancement

**Figure 4:**
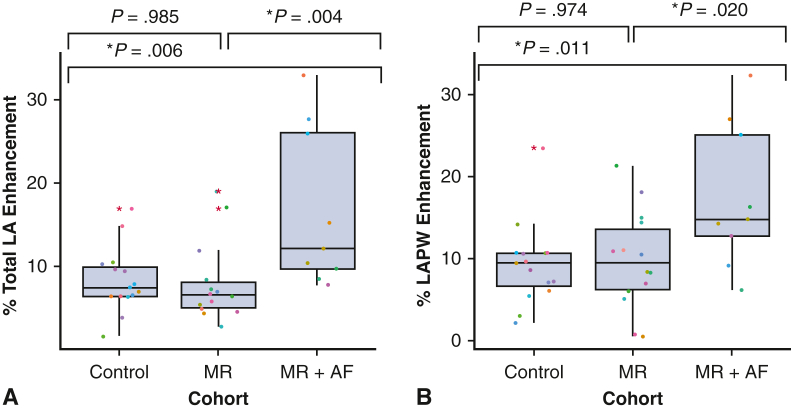
A, Total LA wall enhancement and B, total left atrial posterior wall (LAPW) enhancement. In the *box* and *whiskers* plot, the *lower* and *upper borders* of the *box* represent the *lower* and *upper* quartiles (25th percentile and 75th percentile). The *middle horizontal line* represents the median and the *lower* and *upper whiskers* represent the minimum and maximum values of nonoutliers. *Extra dots* represent outliers. *LA*, Left atrial; *MR*, mitral regurgitation; *AF*, atrial fibrillation.

There was a significant difference in the total LA wall enhancement between controls versus patients with MR + AF (8.3 ± 3.9% vs 16.7 ± 9.6%, *P* = .006) and between patients with lone MR versus patients with MR + AF (8.0 ± 4.8% vs 16.7 ± 9.6%, *P* = .004). There was no difference in LA enhancement between controls and patients with MR (8.3 ± 3.9% vs 8.0 ± 4.8%, *P* = .985; Figure 4, *A*).

### LAPW Regional Enhancement

There was a significant difference in the regional LAPW enhancement between control patients versus patients with MR + AF (9.2 ± 5.1% vs 17.5 ± 8.7%, *P* = .011) and patients with lone MR versus patients with MR + AF (9.8 ± 6.0% vs 17.5 ± 8.7%, *P* = .020). However, there was no difference in LAPW enhancement between controls and patients with MR (9.2 ± 5.1% vs 9.8 ± 6.0%, *P* = .974, Figure 4, *B*).

### LA Wall Enhancement and Indexed LA Volume Correlations

**Figure E2:**
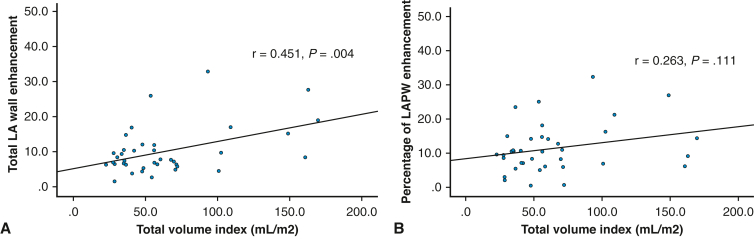
A, Correlation between indexed LA cavitary volume and total LA wall enhancement and B, correlation between LA volume and left atrial posterior wall (*LAPW*). *LA*, Left atrial.

Among all 3 groups, the LA volume correlated with total LA wall enhancement (r = 0.451, *P* = .004; Figure E2, *A*). However, there was no correlation seen between LA volume regional LAPW enhancement (r = 0.263, *P* = .111; Figure E2, *B*).

## Discussion

This study used DE-MRI to quantify LA wall enhancement, a surrogate for fibrosis, in patients with degenerative or calcific MR, with or without a history of AF. Both the total LA wall enhancement and regional LAPW enhancement were found to be significantly greater in patients with MR + AF compared with both controls and patients with lone MR. Further studies will be needed to determine whether duration and classification of AF is significant and/or correlates with the degree and distribution of LA wall fibrosis. However, there was no difference in total LA wall enhancement or regional LAPW enhancement between controls and patients with lone MR. The study also showed a positive correlation between LA volume and total LA wall enhancement. Despite this correlation and a significant increase in wall fibrosis detected between patients with lone MR and patients with MR + AF, there were no significant differences in the LA volumes between these 2 groups. This suggests that atrial fibrosis in patients with MR referred for surgery may be a more important preoperative marker for patients who will develop AF, than simply changes in LA volume alone.

Several studies have used protein molecular analysis on atrial tissue biopsies to show that patients with degenerative MV disease and AF had more fibrosis than patients with MV disease in NSR.^4^, ^5^, ^6^ Our study used noninvasive DE-MRI to show that those with MV disease and AF had more fibrosis than those with lone MR using noninvasive DE-MRI. We have also shown that the LAPW, a clinical important area for arrhythmogenesis for atrial tachyarrhythmias, of patients with MV disease and AF is significantly more enhanced compared with those patients with MV disease in NSR. Similarly in another study, histopathological assessment of the LAPW of those with MV disease and AF had more remodeling than those patients with those with MV disease in NSR.^5^ Overall, few have studied the clinical association between the degree of atrial fibrosis in patients with and without AF who had degenerative MR referred for surgery. The atrial substrate resulting in arrhythmogenesis in these patients remains poorly defined.^3^^,^^11^^,^^22^^,^^23^

There remains controversy as to whether increasing LA volume or worsening atrial fibrosis plays a more important role in the development of AF in patients with degenerative MV disease, as well as the best way to detect these changes. Increases in LA volume and surface area may allow for a critical mass necessary to maintain AF,^24^ whereas increasing fibrosis may impact the conduction properties of the atrial myocardium and provide a more suitable substrate for AF. DE-MRI is the best noninvasive tool to assess LA fibrosis, has been validated by clinical histopathologic assessment of atrial tissue, and allows for precise quantification of LA volume.^8^^,^^12^^,^^13^ Currently, there are 2 comparable and established methods used to quantify LA wall fibrosis with DE-MRI.^25^ The first clinical method, used in our study and the DECAAF trials, quantifies atrial wall fibrosis using a threshold above a certain number of standard deviations, from the mean signal intensity of the myocardium or LA blood pool.^18^^,^^26^ The second method uses an image intensity ratio of 1.2 as the threshold between healthy and fibrotic tissue to normalize the signal intensity of the LA wall to the blood signal intensity.^27^^,^^28^ It is important for clinicians to know that although both methods are similar, they can result in different fibrotic quantifications and fibrosis thresholds depending on the method used, and therefore individual quantification provided by the DE-MRI report may show differences not shown based on these categorization.^25^ In our study, the only patients with Utah class 3 or 4 were in the patients with MR and AF. Although there was no significant difference in fibrosis detected based on the Utah classification (*P* = .065), if we directly compared the average total LA wall and LAPW fibrosis from the individual subjects’ fibrosis reports, there were significant differences.

Clinically, DE-MRI has played a significant role in the diagnostic and prognostic risk stratification of ventricular remodeling from ischemic disease and primary MR.^29^, ^30^, ^31^ Ventricular fibrosis has been proven to be a substrate for reentrant circuits and is predisposing factor for heart failure and sudden cardiac death.^32^^,^^33^ In the last few decades, the use of DE-MRI has been expanded to quantify LA fibrosis in nonvalvular AF and has been used to associate both pre- and postcatheter ablation fibrotic scarring to arrhythmia recurrence.^16^, ^17^, ^18^^,^^34^^,^^35^ Furthermore, even fewer studies have used DE-MRI to assess LA fibrosis in valvular AF.^35^ Identifying these fibrotic patterns using DE-MRI would be valuable in further defining the impact of both MV disease and AF on the degree and distribution of atrial fibrosis.

Our goal was to use these data to help define the arrhythmogenic substrate responsible for AF in patients with MR and a possible threshold of fibrosis that suggest a propensity for future development of AF, and thus clinical recommendation for prophylactic CMP-IV in selected patients. Moreover, the distribution of fibrosis may guide selection of a biatrial versus a left-sided atrial lesion set. Although the DE-MRI protocol is only approved by the Food and Drug Administration for left atrial fibrosis analysis, it would be interesting in future studies to see whether there is any increase in right atrial fibrosis and its distribution to help guide ablation strategies.

Future randomized clinical trials are needed to better understand and define the causal relationship AF induced by degenerative and calcific MR and the development of atrial fibrosis. The limitations of the study are the sample size is small, and there is a heterogeneous clinical presentation of patients with MV disease with and without AF. Despite this, our study remains one of the first and largest in the literature to address this issue in these specific patients, and the enrollment for this prospective cohort study was sufficient to establish significant differences in wall enhancement and volume changes between the groups.

## Conclusions

**Figure 5:**
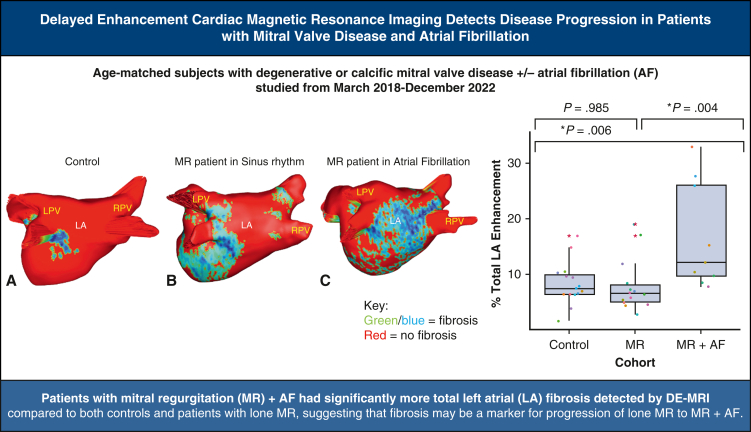
Study methods, results, and implications. *LPV*, Left pulmonary vein; *LA*, left atrial; *RPV*, right pulmonary vein; *MR*, mitral regurgitation; *AF*, atrial fibrillation; *DE-MRI*, delayed-enhancement magnetic resonance imaging.

In conclusion, the main finding in this study was that patients with MR and a history of AF in NSR or AF had significantly greater DE-MRI enhancement than control patients and patients with lone MR in NSR (Figure 5). Moreover, fibrosis was the more determinant factor in defining those patients with AF. The DE-MRI enhancement correlated to larger LA volume, and patients with MR + AF did have a trend toward a greater LA volume compared with patients with lone MR. Future studies, with more enrollment, will be needed to determine whether DE-MRI can be used to determine the need for AF ablation in patients before the onset of the arrhythmia based on a fibrotic threshold and/or to help plan patient-specific ablation lesion sets for patients with MV disease and AF.

### Webcast

You can watch a Webcast of this AATS meeting presentation by going to: https://www.aats.org/resources/delayed-enhancement-cardiac-magnetic-resonance-imaging-can-quantify-disease-progression-in-patients-with-mitral-valve-disease-and-atrial-fibrillation.

**Figure:**
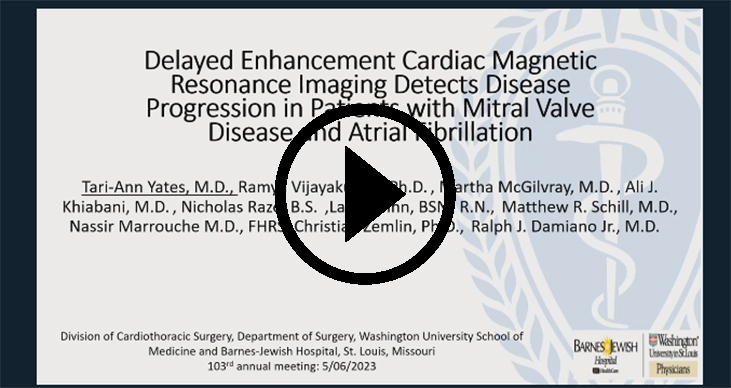

## Conflict of Interest Statement

Ralph J. Damiano, MD, discloses a financial relationship with Medtronic, Inc, AtriCure, Inc, and Edwards Lifesciences. All other authors reported no conflicts of interest.

The *Journal* policy requires editors and reviewers to disclose conflicts of interest and to decline handling or reviewing manuscripts for which they may have a conflict of interest. The editors and reviewers of this article have no conflicts of interest.

## Figures and Tables

**Table E1:** Left atrial fibrosis by DE-MRI and Utah classification of fibrosis

Cohort	Utah class 1 (<10%)	Utah class 2 (10%-20%)	Utah class 3 (20%-30%)	Utah class 4 (>30%)
Controls	11	4	0	0
MR	11	3	0	0
MR + AF in NSR (AF type and duration, mo)	1 (pAF, 2)	2 (pAF, 20) (pAF, 1 d)	1 (pAF, 84)	0
MR + AF in AF (AF type and duration, mo)	2 (1 LSPAF, 18) (1 persistent, 6)	1 (pAF, 4)	1 (pAF, 60)	1 (LSPAF, 21)
Total, n	25	10	2	1

*MR*, Mitral regurgitation; *AF*, atrial fibrillation; *NSR*, normal sinus rhythm; *pAF*, paroxysmal atrial fibrillation; *LSPAF*, longstanding persistent atrial fibrillation; *DE-MRI*, delayed-enhancement magnetic resonance imaging.

## Supplementary Data

Video 1 The authors briefly discuss the study and their main conclusions that fibrosis was most significant in patients with MR + AF and despite volume and enhancement being associated, there was no significant difference in volume seen between patients with lone MR and MR + AF. Therefore, fibrosis detected by DE-MRI may be a marker for progression of lone MR to MR + AF. Video available at: https://www.jtcvs.org/article/S2666-2736(23)00211-5/fulltext.

## References

[1] Yadgir S., Johnson C.O., Aboyans V., Adebayo O.M., Adedoyin R.A., Afarideh M. (2020). Global, regional, and national burden of calcific aortic valve and degenerative mitral valve diseases, 1990-2017. Circulation.

[2] Calkins H., Hindricks G., Cappato R., Kim Y.H., Saad E.B., Aguinaga L. (2017). 2017 HRS/EHRA/ECAS/APHRS/SOLAECE expert consensus statement on catheter and surgical ablation of atrial fibrillation: executive summary. J Arrhythm.

[3] Anne W., Willems R., Roskams T., Sergeant P., Herijgers P., Holemans P. (2005). Matrix metalloproteinases and atrial remodeling in patients with mitral valve disease and atrial fibrillation. Cardiovasc Res.

[4] Zhang P., Wang W., Wang X., Wang X., Song Y., Han Y. (2013). Protein analysis of atrial fibrosis via label-free proteomics in chronic atrial fibrillation patients with mitral valve disease. PLoS One.

[5] Corradi D., Callegari S., Maestri R., Ferrara D., Mangieri D., Alinovi R. (2012). Differential structural remodeling of the left-atrial posterior wall in patients affected by mitral regurgitation with or without persistent atrial fibrillation: a morphological and molecular study. J Cardiovasc Electrophysiol.

[6] Qian Y., Meng J., Tang H., Yang G., Deng Y., Wei D. (2010). Different structural remodelling in atrial fibrillation with different types of mitral valvular diseases. Europace.

[7] Geuzebroek G.S.C., van Amersfoorth S.C.M., Hoogendijk M.G., Kelder J.C., van Hemel N.M., de Bakker J.M.T. (2012). Increased amount of atrial fibrosis in patients with atrial fibrillation secondary to mitral valve disease. J Thorac Cardiovasc Surg.

[8] Boldt A., Wetzel U., Lauschke J., Weigl J., Gummert J., Hindricks G. (2004). Fibrosis in left atrial tissue of patients with atrial fibrillation with and without underlying mitral valve disease. Heart.

[9] Henn M.C., Lancaster T.S., Miller J.R., Sinn L.A., Schuessler R.B., Moon M.R. (2015). Late outcomes after the Cox maze IV procedure for atrial fibrillation. J Thorac Cardiovasc Surg.

[10] Saint L.L., Bailey M.S., Prasad S., Guthrie T.J., Bell J., Moon M.R. (2012). Cox-maze IV results for patients with lone atrial fibrillation versus concomitant mitral disease. Ann Thorac Surg.

[11] Yongjun Q., Huanzhang S., Wenxia Z., Hong T., Xijun X. (2013). Histopathological characteristics and oxidative injury secondary to atrial fibrillation in the left atrial appendages of patients with different forms of mitral valve disease. Cardiovasc Pathol.

[12] Harrison J.L., Jensen H.K., Peel S.A., Chiribiri A., Grøndal A.K., Bloch L.Ø. (2014). Cardiac magnetic resonance and electroanatomical mapping of acute and chronic atrial ablation injury: a histological validation study. Eur Heart J.

[13] McGann C., Akoum N., Patel A., Kholmovski E., Revelo P., Damal K. (2014). Atrial fibrillation ablation outcome is predicted by left atrial remodeling on MRI. Circ Arrhythm Electrophysiol.

[14] Marrouche N.F., Wilber D., Hindricks G., Jais P., Akoum N., Marchlinski F. (2014). Association of atrial tissue fibrosis identified by delayed enhancement MRI and atrial fibrillation catheter ablation. JAMA.

[15] Marrouche N.F., Greene T., Michael Dean J., Kholmovski E.G., Boer L.M., Mansour M. (2021). Efficacy of LGE-MRI-guided fibrosis ablation versus conventional catheter ablation of atrial fibrillation: the DECAAF II trial: study design. J Cardiovasc Electrophysiol.

[16] Robertson J.O., Saint L.L., Leidenfrost J.E., Damiano R.J. (2014). Illustrated techniques for performing the Cox-maze IV procedure through a right mini-thoracotomy. Ann Cardiothorac Surg.

[17] Calkins H., Hindricks G., Cappato R., Kim Y.H., Saad E.B., Aguinaga L. (2017). 2017 HRS/EHRA/ECAS/APHRS/SOLAECE expert consensus statement on catheter and surgical ablation of atrial fibrillation. Heart Rhythm.

[18] Gal P., Marrouche N.F. (2017). Magnetic resonance imaging of atrial fibrosis: redefining atrial fibrillation to a syndrome. Eur Heart J.

[19] Siebermair J., Kholmovski E.G., Marrouche N. (2017). Assessment of left atrial fibrosis by late gadolinium enhancement magnetic resonance imaging: methodology and clinical implications. JACC Clin Electrophysiol.

[20] Kainuma S., Masai T., Yoshitatsu M., Miyagawa S., Yamauchi T., Takeda K. (2011). Advanced left-atrial fibrosis is associated with unsuccessful maze operation for valvular atrial fibrillation. Eur J Cardio Thorac Surg.

[21] Saito T., Tamura K., Uchida D., Saito T., Togashi M., Nitta T. (2007). Histopathological features of the resected left atrial appendage as predictors of recurrence after surgery for atrial fibrillation in valvular heart disease. Circ J.

[22] Byrd G.D., Prasad S.M., Ripplinger C.M., Cassilly T.R., Schuessler R.B., Boineau J.P. (2005). Importance of geometry and refractory period in sustaining atrial fibrillation: testing the critical mass hypothesis. Circulation.

[23] Hopman L.H.G.A., Bhagirath P., Mulder M.J., Eggink I.N., van Rossum A.C., Allaart C.P. (2022). Quantification of left atrial fibrosis by 3D late gadolinium-enhanced cardiac magnetic resonance imaging in patients with atrial fibrillation: impact of different analysis methods. Eur Heart J Cardiovasc Imaging.

[24] Malcolme-Lawes L.C., Juli C., Karim R., Bai W., Quest R., Lim P.B. (2013). Automated analysis of atrial late gadolinium enhancement imaging that correlates with endocardial voltage and clinical outcomes: a 2-center study. Heart Rhythm.

[25] Khurram I.M., Beinart R., Zipunnikov V., Dewire J., Yarmohammadi H., Sasaki T. (2014). Magnetic resonance image intensity ratio, a normalized measure to enable interpatient comparability of left atrial fibrosis. Heart Rhythm.

[26] Benito E.M., Carlosena-Remirez A., Guasch E., Prat-González S., Perea R.J., Figueras R. (2017). Left atrial fibrosis quantification by late gadolinium-enhanced magnetic resonance: a new method to standardize the thresholds for reproducibility. Europace.

[27] Perin E.C., Silva G.V., Sarmento-Leite R., Sousa A.L.S., Howell M., Muthupillai R. (2002). Assessing myocardial viability and infarct transmurality with left ventricular electromechanical mapping in patients with stable coronary artery disease: validation by delayed-enhancement magnetic resonance imaging. Circulation.

[28] Cheong B.Y.C., Muthupillai R., Wilson J.M., Sung A., Huber S., Amin S. (2009). Prognostic significance of delayed-enhancement magnetic resonance imaging: survival of 857 patients with and without left ventricular dysfunction. Circulation.

[29] Gulati A., Jabbour A., Ismail T.F., Guha K., Khwaja J., Raza S. (2013). Association of fibrosis with mortality and sudden cardiac death in patients with nonischemic dilated cardiomyopathy. JAMA.

[30] Bogun F.M., Desjardins B., Good E., Gupta S., Crawford T., Oral H. (2009). Delayed-enhanced magnetic resonance imaging in nonischemic cardiomyopathy. J Am Coll Cardiol.

[31] Wu T.J., Ong J.J., Hwang C., Lee J.J., Fishbein M.C., Czer L. (1998). Characteristics of wave fronts during ventricular fibrillation in human hearts with dilated cardiomyopathy: role of increased fibrosis in the generation of reentry. J Am Coll Cardiol.

[32] Peters D.C., Wylie J.V., Hauser T.H., Kissinger K.V., Botnar R.M., Essebag V. (2007). Detection of pulmonary vein and left atrial scar after catheter ablation with three-dimensional navigator-gated delayed enhancement MR imaging: initial experience. Radiology.

[33] Dickfeld T., Kato R., Zviman M., Lai S., Meininger G., Lardo A.C. (2006). Characterization of radiofrequency ablation lesions with gadolinium-enhanced cardiovascular magnetic resonance imaging. J Am Coll Cardiol.

[34] Ismail A.S., Baghdady Y., Salem M.A., Wahab A.A. (2020). The use of MRI in quantification of the atrial fibrosis in patients with rheumatic mitral disease. Egyptian J Radiol Nucl Med.

[35] Zhu D., Wu Z., van der Geest R.J., Luo Y., Sun J., Jiang J. (2015). Accuracy of late gadolinium enhancement-magnetic resonance imaging in the measurement of left atrial substrate remodeling in patients with rheumatic mitral valve disease and persistent atrial fibrillation. Int Heart J.

