# Supplementary figures and images for: Delayed-enhancement cardiac magnetic resonance imaging detects disease progression in patients with mitral valve disease and atrial fibrillation
Source: JTCVS Open. 2023 Aug 17;16:292–302. doi: 10.1016/j.xjon.2023.07.024 (PMC10774962; doi:10.1016/j.xjon.2023.07.024)

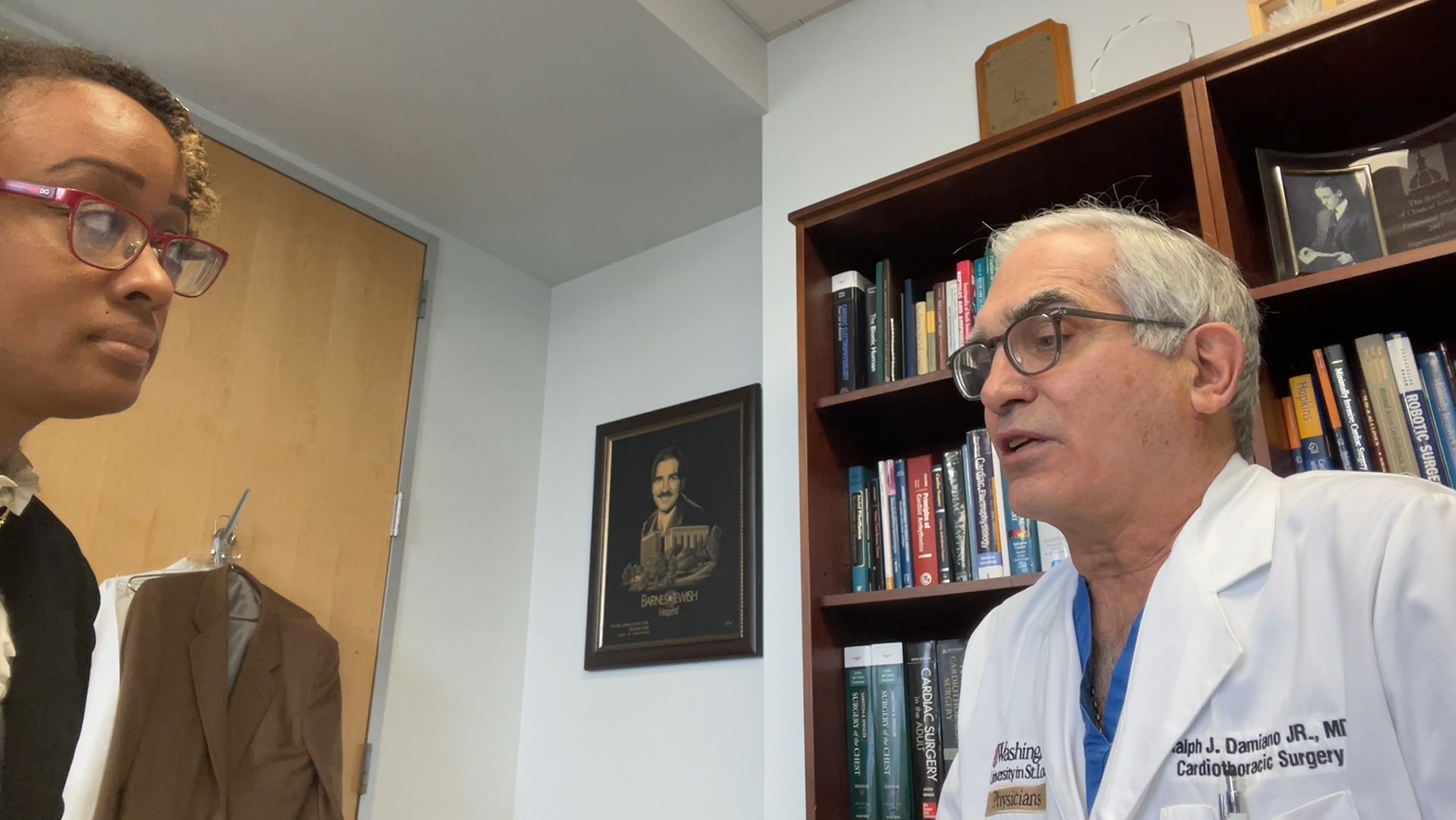

Supplement: Supplementary file 2 [file fx3.jpg]

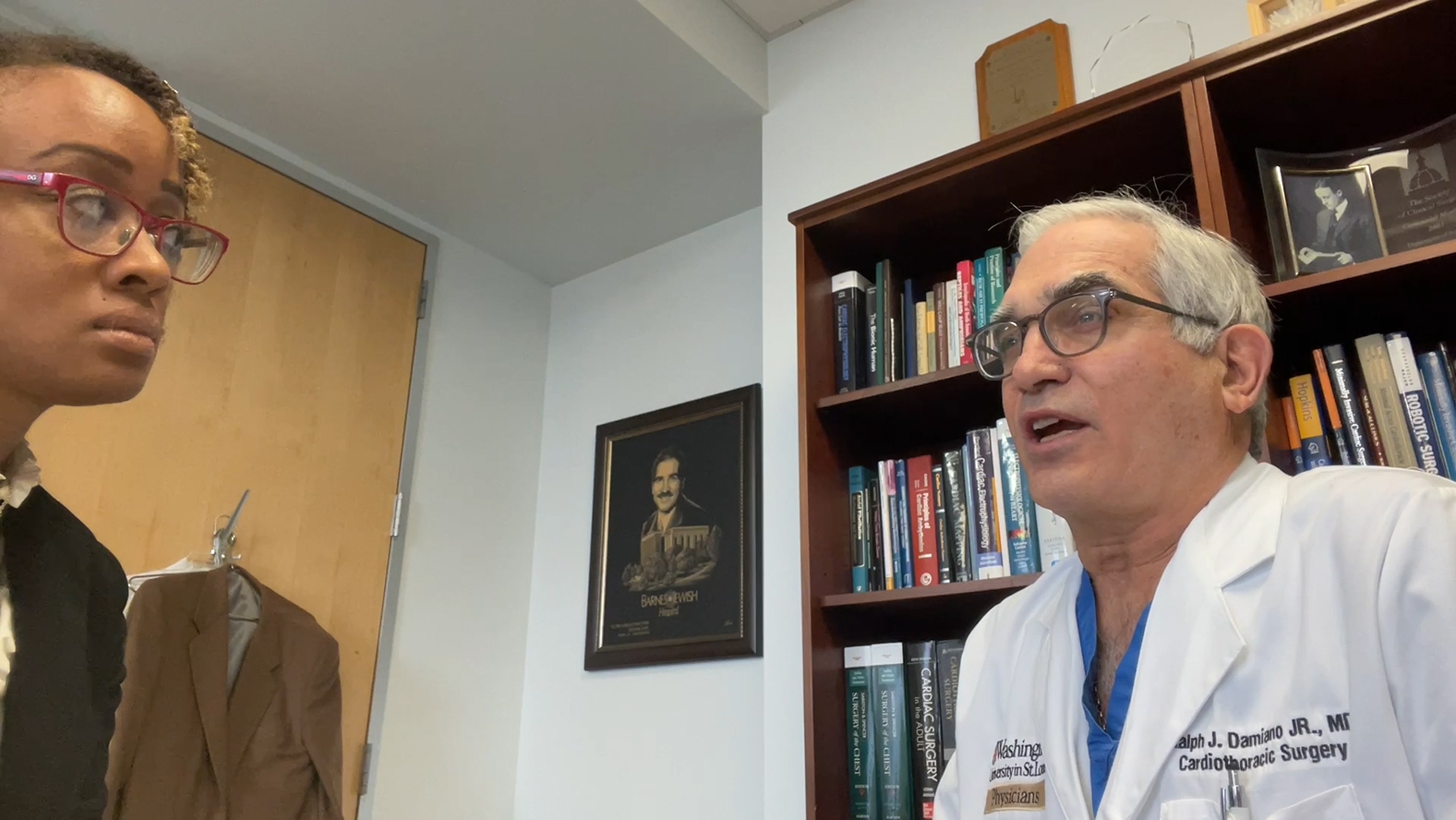

Supplement: Supplementary file 4 [file fx4.jpg]

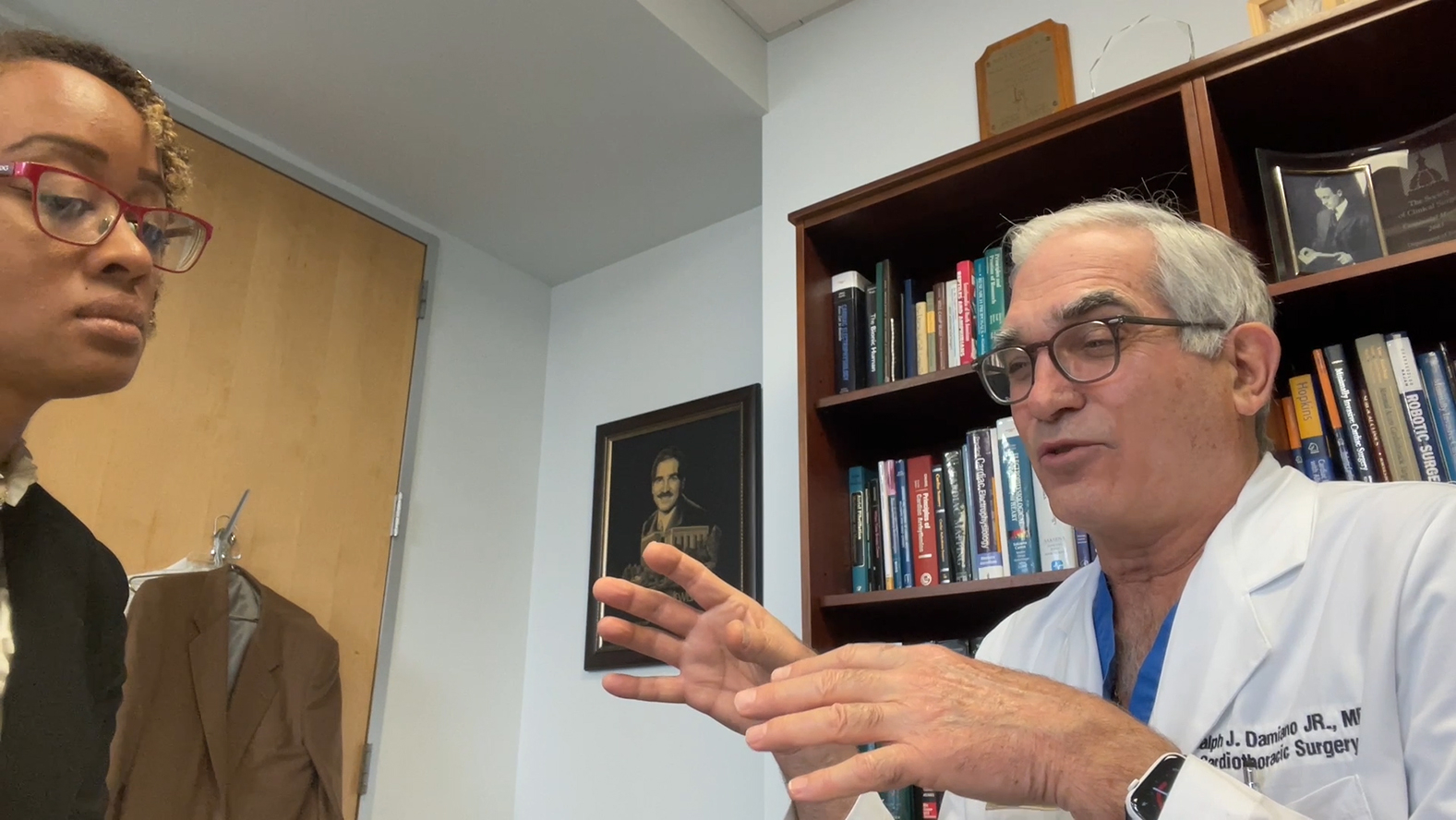

Supplement: Supplementary file 6 [file fx5.jpg]

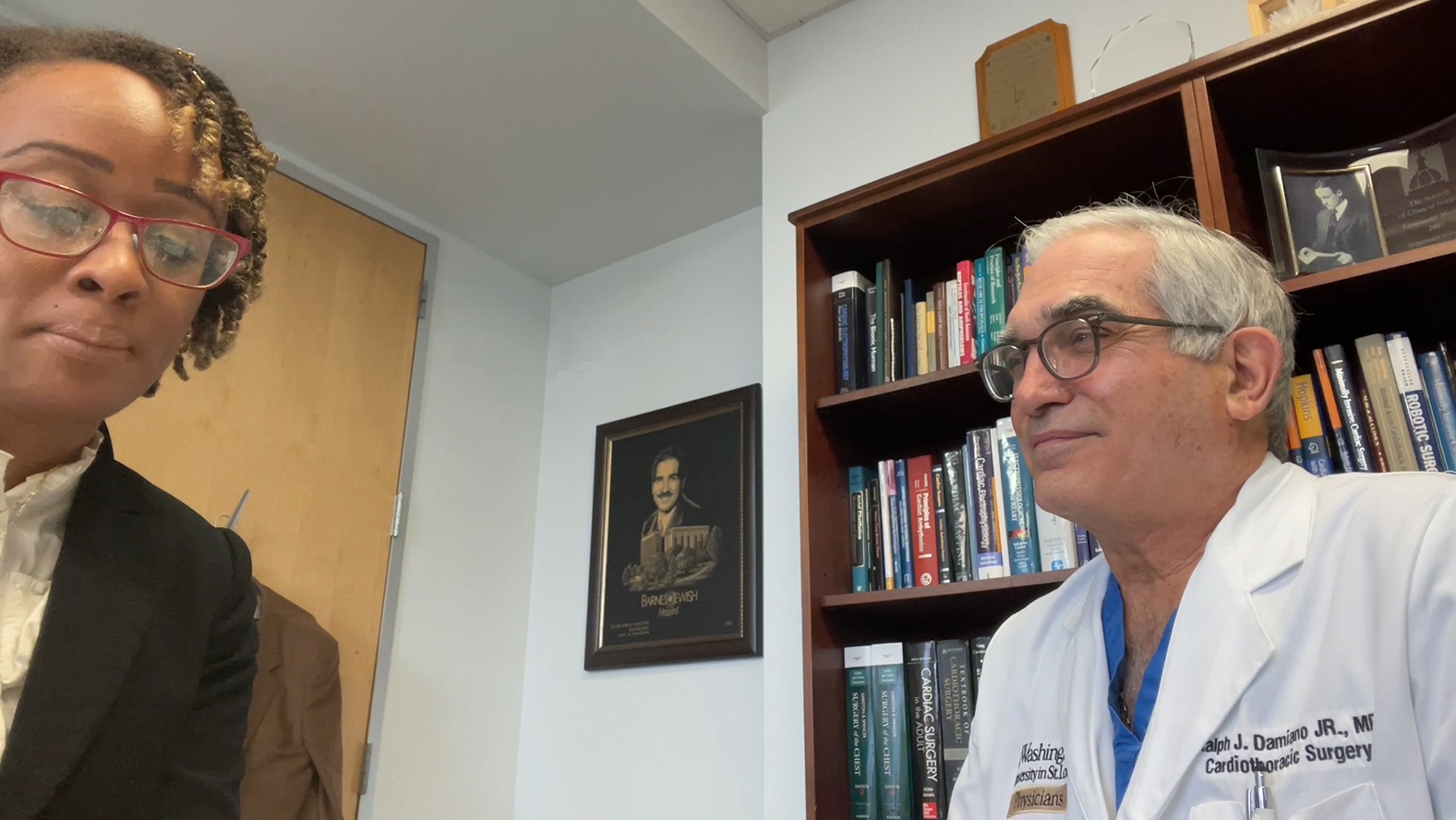

Supplement: Video 1 — The authors briefly discuss the study and their main conclusions that fibrosis was most significant in patients with MR + AF and despite volume and enhancement being associated, there was no significant difference in volume seen between patients with lone MR and MR + AF. Therefore, fibrosis detected by DE-MRI may be a marker for progression of lone MR to MR + AF. Video available at: https://www.jtcvs.org/article/S2666-2736(23)00211-5/fulltext. [file fx6.jpg]
